# 5-Chloro-3-cyclo­hexyl­sulfinyl-2,7-dimethyl-1-benzofuran

**DOI:** 10.1107/S1600536811033095

**Published:** 2011-08-27

**Authors:** Hong Dae Choi, Pil Ja Seo, Uk Lee

**Affiliations:** a Department of Chemistry, Dongeui University, San 24 Kaya-dong Busanjin-gu, Busan 614-714, Republic of Korea; b Department of Chemistry, Pukyong National University, 599-1 Daeyeon 3-dong, Nam-gu, Busan 608-737, Republic of Korea

## Abstract

In the title compound, C_16_H_19_ClO_2_S, the cyclo­hexyl ring adopts a chair conformation and the aryl­sulfinyl unit is positioned equatorial relative to the cyclo­hexyl group. The least-squares plane through all six C atoms of the cyclo­hexyl ring makes a dihedral angle of 74.80 (6)° with the mean plane of the benzofuran fragment. In the crystal, mol­ecules are linked through inter­molecular C—H⋯O hydrogen bonds.

## Related literature

For the pharmacological activity of benzofuran compounds, see: Aslam *et al.* (2009 ▶); Galal *et al.* (2009 ▶); Khan *et al.* (2005 ▶). For natural products with benzofuran rings, see: Akgul & Anil (2003 ▶); Soekamto *et al.* (2003 ▶). For structural studies of related 3-cyclo­hexyl­sulfinyl-5-halo-2-methyl-1-benzofuran derivatives, see: Choi *et al.* (2011**a* ▶,*b* ▶,c* ▶).
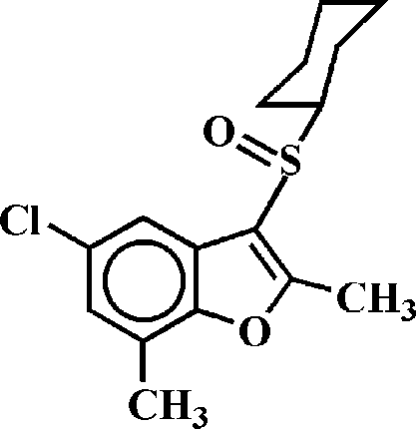


## Experimental

### 

#### Crystal data

C_16_H_19_ClO_2_S
*M* *_r_* = 310.82
Triclinic, 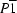

*a* = 5.7480 (3) Å
*b* = 11.6838 (5) Å
*c* = 12.2551 (5) Å
α = 70.076 (3)°
β = 77.244 (2)°
γ = 83.413 (3)°
*V* = 753.98 (6) Å^3^
*Z* = 2
Mo *K*α radiation
μ = 0.39 mm^−1^
*T* = 173 K
0.33 × 0.18 × 0.14 mm

#### Data collection

Bruker SMART APEXII CCD diffractometer
Absorption correction: multi-scan (*SADABS*; Bruker, 2009 ▶) *T*_min_ = 0.883, *T*_max_ = 0.948
13573 measured reflections
3487 independent reflections
2752 reflections with *I* > 2σ(*I*)
*R*_int_ = 0.049

#### Refinement

*R*[*F* ^2^ > 2σ(*F* ^2^)] = 0.044
*wR*(*F* ^2^) = 0.116
*S* = 1.06
3487 reflections
183 parameters
H-atom parameters constrained
Δρ_max_ = 0.44 e Å^−3^
Δρ_min_ = −0.27 e Å^−3^

### 

Data collection: *APEX2* (Bruker, 2009 ▶); cell refinement: *SAINT* (Bruker, 2009 ▶); data reduction: *SAINT*; program(s) used to solve structure: *SHELXS97* (Sheldrick, 2008 ▶); program(s) used to refine structure: *SHELXL97* (Sheldrick, 2008 ▶); molecular graphics: *ORTEP-3* (Farrugia, 1997 ▶) and *DIAMOND* (Brandenburg, 1998 ▶); software used to prepare material for publication: *SHELXL97*.

## Supplementary Material

Crystal structure: contains datablock(s) global, I. DOI: 10.1107/S1600536811033095/gw2105sup1.cif

Structure factors: contains datablock(s) I. DOI: 10.1107/S1600536811033095/gw2105Isup2.hkl

Supplementary material file. DOI: 10.1107/S1600536811033095/gw2105Isup3.cml

Additional supplementary materials:  crystallographic information; 3D view; checkCIF report

## Figures and Tables

**Table 1:** Hydrogen-bond geometry (Å, °)

*D*—H⋯*A*	*D*—H	H⋯*A*	*D*⋯*A*	*D*—H⋯*A*
C11—H11⋯O2^i^	1.00	2.31	3.276 (2)	163

Symmetry code: (i) 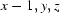.

**Fig. 1.**
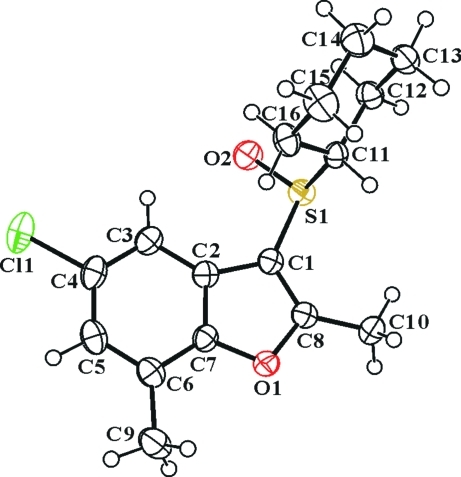
The molecular structure of the title compound with the atom numbering scheme. Displacement ellipsoids are drawn at the 50% probability level. H atoms are presented as a small spheres of arbitrary radius.

**Fig. 2.**
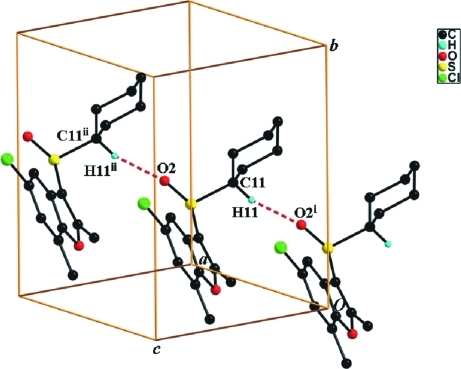
A view of the C—H···O hydrogen bonds (dotted lines) in the crystal structure of the title compound. [Symmetry codes: (i) x - 1, y, z; (ii) x + 1, y, z.]

## supplementary crystallographic information

### Comment

Recently, many compounds containing a benzofuran skeleton have drawn much attention owing to their significant pharmacological properties such as antibacterial and antifungal, antitumor and antiviral, and antimicrobial activities (Aslam *et al.*, 2009, Galal *et al.*, 2009, Khan *et al.*, 2005). These compounds occur in a wide range of natural products (Akgul & Anil, 2003; Soekamto *et al.*, 2003). As a part of our ongoing study of the substituent effect on the solid state structures of 3-cyclohexylsulfinyl-5-halo-2-methyl-1-benzofuran analogues (Choi *et al.*, 2011**a*,*b*,c*), we report herein the crystal structure of the title compound.

In the title molecule (Fig. 1), the benzofuran unit is essentially planar, with a mean deviation of 0.009 (1) Å from the least-squares plane defined by the nine constituent atoms. The cyclohexyl ring is in the chair form. The least-squares plane through all six C atoms of the cyclohexyl ring makes a dihedral angle of 74.80 (6)°° with the mean plane of the benzofuran fragment. The molecular packing (Fig. 2) is stabilized by intermolecular C—H···O hydrogen bonds between a cyclohexyl H atom and the O atom of the sulfinyl group (Table 1; C11—H11···O2^i^).

### Experimental

77% 3-chloroperoxybenzoic acid (247 mg, 1.1 mmol) was added in small portions to a stirred solution of 5-chloro-3-cyclohexylsulfanyl-2,7-dimethyl-1-benzofuran (324 mg, 1.1 mmol) in dichloromethane (40 mL) at 273 K. After being stirred at room temperature for 4h, the mixture was washed with saturated sodium bicarbonate solution and the organic layer was separated, dried over magnesium sulfate, filtered and concentrated at reduced pressure. The residue was purified by column chromatography (hexane–ethyl acetate, 1:1 v/v) to afford the title compound as a colorless solid [yield 73%, m.p. 405–406 K; *R*_f_ = 0.45 (hexane–ethyl acetate, 1:1 v/v)]. Single crystals suitable for X-ray diffraction were prepared by slow evaporation of a solution of the title compound in ethyl acetate at room temperature.

### Refinement

All H atoms were positioned geometrically and refined using a riding model, with C—H = 0.95 Å  for aryl, 1.00 Å for methine, 0.99 Å for methylene and 0.98 Å for methyl H atoms, respectively. *U*_iso_(H)  = 1.2*U*_eq_(C)  for aryl,  methine,  methylene,  and 1.5*U*_eq_(C) for methyl H atoms.

### Crystal data

**Table:**

C_16_H_19_ClO_2_S	*Z* = 2
*M**_r_* = 310.82	*F*(000) = 328
Triclinic, *P*1	*D*_x_ = 1.369 Mg m^−^^3^
Hall symbol: -P 1	Mo *K*α radiation, λ = 0.71073 Å
*a* = 5.7480 (3) Å	Cell parameters from 4179 reflections
*b* = 11.6838 (5) Å	θ = 3.0–27.4°
*c* = 12.2551 (5) Å	µ = 0.39 mm^−^^1^
α = 70.076 (3)°	*T* = 173 K
β = 77.244 (2)°	Block, colourless
γ = 83.413 (3)°	0.33 × 0.18 × 0.14 mm
*V* = 753.98 (6) Å^3^	

### Data collection

**Table:**

Bruker SMART APEXII CCD diffractometer	3487 independent reflections
Radiation source: rotating anode	2752 reflections with *I* > 2σ(*I*)
graphite multilayer	*R*_int_ = 0.049
Detector resolution: 10.0 pixels mm^-1^	θ_max_ = 27.7°, θ_min_ = 1.8°
φ and ω scans	*h* = −7→6
Absorption correction: multi-scan (*SADABS*; Bruker, 2009)	*k* = −15→15
*T*_min_ = 0.883, *T*_max_ = 0.948	*l* = −15→15
13573 measured reflections	

### Refinement

**Table:**

Refinement on *F*^2^	Primary atom site location: structure-invariant direct methods
Least-squares matrix: full	Secondary atom site location: difference Fourier map
*R*[*F*^2^ > 2σ(*F*^2^)] = 0.044	Hydrogen site location: difference Fourier map
*wR*(*F*^2^) = 0.116	H-atom parameters constrained
*S* = 1.06	*w* = 1/[σ^2^(*F*_o_^2^) + (0.0491*P*)^2^ + 0.2498*P*] where *P* = (*F*_o_^2^ + 2*F*_c_^2^)/3
3487 reflections	(Δ/σ)_max_ < 0.001
183 parameters	Δρ_max_ = 0.44 e Å^−^^3^
0 restraints	Δρ_min_ = −0.27 e Å^−^^3^

### Special details

**Table:**

Geometry. All esds (except the esd in the dihedral angle between two l.s. planes) are estimated using the full covariance matrix. The cell esds are taken into account individually in the estimation of esds in distances, angles and torsion angles; correlations between esds in cell parameters are only used when they are defined by crystal symmetry. An approximate (isotropic) treatment of cell esds is used for estimating esds involving l.s. planes.
Refinement. Refinement of F^2^ against ALL reflections. The weighted R-factor wR and goodness of fit S are based on F^2^, conventional R-factors R are based on F, with F set to zero for negative F^2^. The threshold expression of F^2^ > 2sigma(F^2^) is used only for calculating R-factors(gt) etc. and is not relevant to the choice of reflections for refinement. R-factors based on F^2^ are statistically about twice as large as those based on F, and R- factors based on ALL data will be even larger.

### Fractional atomic coordinates and isotropic or equivalent isotropic displacement parameters (Å^2^)

**Table:**

	*x*	*y*	*z*	*U*_iso_*/*U*_eq_	
Cl1	1.26205 (10)	0.19475 (6)	0.06775 (5)	0.05045 (18)	
S1	0.47811 (9)	0.36573 (4)	0.41383 (4)	0.03095 (15)	
O1	0.3493 (2)	0.05713 (11)	0.37764 (11)	0.0314 (3)	
O2	0.7264 (3)	0.40766 (12)	0.37025 (14)	0.0415 (4)	
C1	0.4738 (4)	0.23324 (15)	0.37493 (16)	0.0288 (4)	
C2	0.6529 (3)	0.18767 (16)	0.29578 (16)	0.0288 (4)	
C3	0.8708 (4)	0.22616 (17)	0.22279 (17)	0.0333 (4)	
H3	0.9363	0.2995	0.2171	0.040*	
C4	0.9868 (4)	0.15224 (19)	0.15917 (17)	0.0365 (5)	
C5	0.8937 (4)	0.04503 (18)	0.16450 (17)	0.0368 (5)	
H5	0.9814	−0.0019	0.1180	0.044*	
C6	0.6771 (4)	0.00549 (17)	0.23564 (17)	0.0338 (4)	
C7	0.5656 (4)	0.07938 (16)	0.30115 (16)	0.0295 (4)	
C8	0.2989 (4)	0.15212 (15)	0.42236 (16)	0.0296 (4)	
C9	0.5649 (4)	−0.10708 (18)	0.2428 (2)	0.0449 (5)	
H9A	0.6628	−0.1432	0.1855	0.067*	
H9B	0.4045	−0.0858	0.2248	0.067*	
H9C	0.5539	−0.1659	0.3228	0.067*	
C10	0.0724 (4)	0.14949 (17)	0.50709 (17)	0.0347 (4)	
H10A	0.0625	0.2173	0.5381	0.052*	
H10B	0.0653	0.0722	0.5725	0.052*	
H10C	−0.0614	0.1573	0.4671	0.052*	
C11	0.2917 (3)	0.46699 (15)	0.31664 (15)	0.0271 (4)	
H11	0.1319	0.4306	0.3381	0.033*	
C12	0.3878 (4)	0.48526 (18)	0.18677 (17)	0.0365 (5)	
H12A	0.3985	0.4062	0.1726	0.044*	
H12B	0.5503	0.5168	0.1640	0.044*	
C13	0.2246 (5)	0.5751 (2)	0.11125 (18)	0.0458 (6)	
H13A	0.2956	0.5905	0.0265	0.055*	
H13B	0.0680	0.5388	0.1275	0.055*	
C14	0.1878 (5)	0.69445 (19)	0.13643 (18)	0.0436 (5)	
H14A	0.0734	0.7484	0.0899	0.052*	
H14B	0.3414	0.7354	0.1117	0.052*	
C15	0.0929 (4)	0.67408 (17)	0.26706 (18)	0.0374 (5)	
H15A	−0.0679	0.6405	0.2899	0.045*	
H15B	0.0777	0.7531	0.2818	0.045*	
C16	0.2574 (4)	0.58673 (16)	0.34240 (17)	0.0337 (4)	
H16A	0.4139	0.6234	0.3250	0.040*	
H16B	0.1876	0.5717	0.4272	0.040*	

### Atomic displacement parameters (Å^2^)

**Table:**

	*U*^11^	*U*^22^	*U*^33^	*U*^12^	*U*^13^	*U*^23^
Cl1	0.0289 (3)	0.0773 (4)	0.0411 (3)	−0.0036 (3)	−0.0002 (3)	−0.0178 (3)
S1	0.0310 (3)	0.0328 (2)	0.0332 (3)	−0.0023 (2)	−0.0108 (2)	−0.01293 (18)
O1	0.0281 (8)	0.0294 (6)	0.0369 (7)	−0.0023 (6)	−0.0058 (6)	−0.0110 (5)
O2	0.0292 (8)	0.0430 (7)	0.0580 (9)	−0.0037 (7)	−0.0144 (8)	−0.0194 (7)
C1	0.0275 (10)	0.0299 (8)	0.0300 (9)	0.0001 (8)	−0.0086 (9)	−0.0095 (7)
C2	0.0259 (10)	0.0321 (8)	0.0301 (9)	0.0006 (8)	−0.0111 (9)	−0.0092 (7)
C3	0.0290 (11)	0.0382 (9)	0.0328 (10)	−0.0039 (9)	−0.0088 (9)	−0.0092 (8)
C4	0.0253 (11)	0.0504 (11)	0.0307 (10)	0.0004 (9)	−0.0069 (9)	−0.0088 (8)
C5	0.0347 (12)	0.0458 (11)	0.0327 (10)	0.0108 (10)	−0.0116 (10)	−0.0169 (8)
C6	0.0338 (12)	0.0351 (9)	0.0351 (10)	0.0058 (9)	−0.0129 (10)	−0.0132 (8)
C7	0.0251 (10)	0.0323 (8)	0.0305 (9)	−0.0005 (8)	−0.0071 (9)	−0.0084 (7)
C8	0.0292 (11)	0.0296 (8)	0.0311 (9)	0.0002 (8)	−0.0095 (9)	−0.0097 (7)
C9	0.0470 (14)	0.0391 (10)	0.0557 (13)	0.0044 (10)	−0.0139 (12)	−0.0239 (10)
C10	0.0303 (11)	0.0391 (10)	0.0339 (10)	−0.0053 (9)	−0.0029 (9)	−0.0116 (8)
C11	0.0227 (10)	0.0315 (8)	0.0293 (9)	−0.0021 (8)	−0.0060 (8)	−0.0118 (7)
C12	0.0382 (12)	0.0432 (10)	0.0306 (9)	0.0054 (9)	−0.0069 (10)	−0.0174 (8)
C13	0.0556 (16)	0.0515 (12)	0.0327 (10)	0.0089 (11)	−0.0156 (11)	−0.0160 (9)
C14	0.0481 (15)	0.0425 (11)	0.0357 (11)	0.0048 (10)	−0.0107 (11)	−0.0074 (8)
C15	0.0389 (13)	0.0337 (9)	0.0399 (11)	0.0027 (9)	−0.0060 (10)	−0.0146 (8)
C16	0.0378 (12)	0.0337 (9)	0.0329 (10)	−0.0010 (9)	−0.0061 (9)	−0.0157 (8)

### Geometric parameters (Å, °)

**Table:**

Cl1—C4	1.739 (2)	C10—H10A	0.9800
S1—O2	1.4860 (15)	C10—H10B	0.9800
S1—C1	1.7723 (18)	C10—H10C	0.9800
S1—C11	1.8107 (19)	C11—C12	1.514 (3)
O1—C7	1.373 (2)	C11—C16	1.519 (2)
O1—C8	1.374 (2)	C11—H11	1.0000
C1—C8	1.354 (3)	C12—C13	1.528 (3)
C1—C2	1.438 (3)	C12—H12A	0.9900
C2—C7	1.390 (2)	C12—H12B	0.9900
C2—C3	1.390 (3)	C13—C14	1.510 (3)
C3—C4	1.377 (3)	C13—H13A	0.9900
C3—H3	0.9500	C13—H13B	0.9900
C4—C5	1.394 (3)	C14—C15	1.517 (3)
C5—C6	1.379 (3)	C14—H14A	0.9900
C5—H5	0.9500	C14—H14B	0.9900
C6—C7	1.385 (3)	C15—C16	1.520 (3)
C6—C9	1.498 (3)	C15—H15A	0.9900
C8—C10	1.471 (3)	C15—H15B	0.9900
C9—H9A	0.9800	C16—H16A	0.9900
C9—H9B	0.9800	C16—H16B	0.9900
C9—H9C	0.9800		
			
O2—S1—C1	106.29 (9)	H10A—C10—H10C	109.5
O2—S1—C11	108.27 (9)	H10B—C10—H10C	109.5
C1—S1—C11	98.88 (8)	C12—C11—C16	111.89 (15)
C7—O1—C8	106.36 (14)	C12—C11—S1	113.85 (13)
C8—C1—C2	107.62 (16)	C16—C11—S1	107.54 (12)
C8—C1—S1	124.13 (15)	C12—C11—H11	107.8
C2—C1—S1	128.16 (14)	C16—C11—H11	107.8
C7—C2—C3	119.49 (17)	S1—C11—H11	107.8
C7—C2—C1	104.58 (17)	C11—C12—C13	110.31 (17)
C3—C2—C1	135.92 (17)	C11—C12—H12A	109.6
C4—C3—C2	116.40 (18)	C13—C12—H12A	109.6
C4—C3—H3	121.8	C11—C12—H12B	109.6
C2—C3—H3	121.8	C13—C12—H12B	109.6
C3—C4—C5	123.1 (2)	H12A—C12—H12B	108.1
C3—C4—Cl1	118.46 (16)	C14—C13—C12	111.64 (18)
C5—C4—Cl1	118.48 (17)	C14—C13—H13A	109.3
C6—C5—C4	121.56 (19)	C12—C13—H13A	109.3
C6—C5—H5	119.2	C14—C13—H13B	109.3
C4—C5—H5	119.2	C12—C13—H13B	109.3
C5—C6—C7	114.61 (18)	H13A—C13—H13B	108.0
C5—C6—C9	124.04 (19)	C13—C14—C15	110.96 (17)
C7—C6—C9	121.34 (19)	C13—C14—H14A	109.4
O1—C7—C6	124.32 (17)	C15—C14—H14A	109.4
O1—C7—C2	110.81 (16)	C13—C14—H14B	109.4
C6—C7—C2	124.84 (19)	C15—C14—H14B	109.4
C1—C8—O1	110.61 (17)	H14A—C14—H14B	108.0
C1—C8—C10	132.59 (17)	C14—C15—C16	111.22 (17)
O1—C8—C10	116.78 (15)	C14—C15—H15A	109.4
C6—C9—H9A	109.5	C16—C15—H15A	109.4
C6—C9—H9B	109.5	C14—C15—H15B	109.4
H9A—C9—H9B	109.5	C16—C15—H15B	109.4
C6—C9—H9C	109.5	H15A—C15—H15B	108.0
H9A—C9—H9C	109.5	C11—C16—C15	110.08 (16)
H9B—C9—H9C	109.5	C11—C16—H16A	109.6
C8—C10—H10A	109.5	C15—C16—H16A	109.6
C8—C10—H10B	109.5	C11—C16—H16B	109.6
H10A—C10—H10B	109.5	C15—C16—H16B	109.6
C8—C10—H10C	109.5	H16A—C16—H16B	108.2
			
O2—S1—C1—C8	163.69 (16)	C3—C2—C7—O1	−179.98 (16)
C11—S1—C1—C8	−84.21 (17)	C1—C2—C7—O1	−0.2 (2)
O2—S1—C1—C2	−12.42 (19)	C3—C2—C7—C6	−1.9 (3)
C11—S1—C1—C2	99.67 (18)	C1—C2—C7—C6	177.87 (18)
C8—C1—C2—C7	0.8 (2)	C2—C1—C8—O1	−1.2 (2)
S1—C1—C2—C7	177.46 (14)	S1—C1—C8—O1	−177.97 (13)
C8—C1—C2—C3	−179.5 (2)	C2—C1—C8—C10	−179.5 (2)
S1—C1—C2—C3	−2.8 (3)	S1—C1—C8—C10	3.7 (3)
C7—C2—C3—C4	0.2 (3)	C7—O1—C8—C1	1.0 (2)
C1—C2—C3—C4	−179.4 (2)	C7—O1—C8—C10	179.65 (16)
C2—C3—C4—C5	0.9 (3)	O2—S1—C11—C12	51.25 (15)
C2—C3—C4—Cl1	−179.20 (14)	C1—S1—C11—C12	−59.27 (15)
C3—C4—C5—C6	−0.5 (3)	O2—S1—C11—C16	−73.30 (14)
Cl1—C4—C5—C6	179.60 (15)	C1—S1—C11—C16	176.18 (13)
C4—C5—C6—C7	−1.0 (3)	C16—C11—C12—C13	−55.8 (2)
C4—C5—C6—C9	178.37 (19)	S1—C11—C12—C13	−178.01 (14)
C8—O1—C7—C6	−178.57 (18)	C11—C12—C13—C14	55.1 (2)
C8—O1—C7—C2	−0.5 (2)	C12—C13—C14—C15	−55.6 (3)
C5—C6—C7—O1	−179.95 (17)	C13—C14—C15—C16	56.4 (3)
C9—C6—C7—O1	0.6 (3)	C12—C11—C16—C15	56.7 (2)
C5—C6—C7—C2	2.2 (3)	S1—C11—C16—C15	−177.55 (14)
C9—C6—C7—C2	−177.18 (19)	C14—C15—C16—C11	−56.5 (2)

### Hydrogen-bond geometry (Å, °)

**Table:**

*D*—H···*A*	*D*—H	H···*A*	*D*···*A*	*D*—H···*A*
C11—H11···O2^i^	1.00	2.31	3.276 (2)	163.

Symmetry codes: (i) *x*−1, *y*, *z*.
